# Pharmacological preconditioning with gemfibrozil preserves cardiac function after heart transplantation

**DOI:** 10.1038/s41598-017-14587-3

**Published:** 2017-10-27

**Authors:** Kálmán Benke, Csaba Mátyás, Alex Ali Sayour, Attila Oláh, Balázs Tamás Németh, Mihály Ruppert, Gábor Szabó, Gábor Kökény, Eszter Mária Horváth, István Hartyánszky, Zoltán Szabolcs, Béla Merkely, Tamás Radovits

**Affiliations:** 1 Heart and Vascular Center, Semmelweis University, Budapest, Hungary; 2 Department of Cardiac Surgery, University of Heidelberg, Heidelberg, Germany; 3 Department of Pathophysiology, Semmelweis University, Budapest, Hungary; 4 Department of Physiology, Semmelweis University, Budapest, Hungary

## Abstract

While heart transplantation (HTX) is the definitive therapy of heart failure, donor shortage is emerging. Pharmacological activation of soluble guanylate cyclase (sGC) and increased cGMP-signalling have been reported to have cardioprotective properties. Gemfibrozil has recently been shown to exert sGC activating effects *in vitro*. We aimed to investigate whether pharmacological preconditioning of donor hearts with gemfibrozil could protect against ischemia/reperfusion injury and preserve myocardial function in a heterotopic rat HTX model. Donor Lewis rats received p.o. gemfibrozil (150 mg/kg body weight) or vehicle for 2 days. The hearts were explanted, stored for 1 h in cold preservation solution, and heterotopically transplanted. 1 h after starting reperfusion, left ventricular (LV) pressure-volume relations and coronary blood flow (CBF) were assessed to evaluate early post-transplant graft function. After 1 h reperfusion, LV contractility, active relaxation and CBF were significantly (p < 0.05) improved in the gemfibrozil pretreated hearts compared to that of controls. Additionally, gemfibrozil treatment reduced nitro-oxidative stress and apoptosis, and improved cGMP-signalling in HTX. Pharmacological preconditioning with gemfibrozil reduces ischemia/reperfusion injury and preserves graft function in a rat HTX model, which could be the consequence of enhanced myocardial cGMP-signalling. Gemfibrozil might represent a useful tool for cardioprotection in the clinical setting of HTX surgery soon.

## Introduction

Although there is a rapid evolution of mechanical circulatory support, heart transplantation (HTX) is still the gold standard definitive therapy of end-stage heart failure. Ischemia/reperfusion injury is one of the major determinants of primary graft failure and long-term outcome in HTX^1^. Possible prevention of such an injury can be achieved by a professional transplant team with efficient logistics, developing surgical techniques and novel cardioplegic solutions^2^. On the other hand, pharmacological preconditioning of the donor heart before explantation is a possible approach to reduce ischemia/reperfusion damage of the graft^3^.

During the reperfusion phase, the myocardium of the implanted graft suffers from biochemical and metabolic alterations, including generation of reactive oxygen species (ROS), intracellular calcium overload, energy depletion and acidosis^4^. There were several attempts to reduce these biochemical changes, however the most promising novel therapeutic tools modulate the nitric oxide (NO)/soluble guanylate cyclase (sGC)/cyclic guanosine monophosphate (cGMP)/protein kinase G (PKG) pathway^5^.

Under physiological circumstances, NO binds to the haem moiety of sGC, which results in the production of cGMP. Furthermore cGMP activates PKG, that phosphorylates the important effectors which play key roles in the regulation of vasodilation, inhibition of platelet aggregation and vascular smooth muscle proliferation^6^. In recent studies, cGMP signalling has been implicated in cardioprotective mechanisms in different disease conditions^7^.

Increased oxidative stress during ischemia/reperfusion leads to the oxidation of sGC, thereby impairing its activity and responsiveness to endogenous NO, which mechanism could result in the deterioration of the cGMP signalling cascade^7^.

According to recent experimental data pharmacological activation of sGC could be a promising tool to prevent myocardial oxidative damage^8^.

Cinaciguat (BAY 58-2667), a member of the novel drug family of sGC activators has been extensively investigated and shown to reduce ischemia/reperfusion injury in experimental models of myocardial infarction and HTX^1,8^. In spite of the promising results of the pre-clinical studies, the clinical development of cinaciguat for the indication of acute decompensated heart failure had to be prematurely terminated due to its hypotensive side effect after acute iv. application^9^.

Gemfibrozil (GEM) is one of the fibrate drugs, which has been used in the clinical routine for decades for the management of combined dyslipidaemia^10^. The mechanism of action of this drug is the activation of the peroxisome proliferator-activated receptor-alpha (PPARα)^11^, which is a nuclear receptor responsible for the metabolism of carbohydrates and fats^12^. Interestingly Sharina *et al*.^13^ described an existing side effect of GEM in 2015, i.e. activation of the sGC in an *in vitro* setup. Although sporadic literature data exist^14–16^, the possible cardioprotective effects of GEM have been poorly investigated.

We aimed at evaluating the potential cardioprotective effects of GEM in a clinically relevant, well established rat model of HTX^3,17^.

## Results

### Effect of gemfibrozil treatment on routine biochemical laboratory parameters and hemodynamic indices of donor rats

Gemfibrozil in a dose of 150 mg/kg body weight had no significant (p < 0.05) side effect on the kidney and liver parameters in our non-transplanted rats, whereas the total, HDL and LDL cholesterol were significantly (p < 0.05) decreased (Table 1). Trigliceride plasma level was also elevated in the Gem-nHTX group. Cardiac performance in both systole and diastole, as well as mean arterial pressure of the gemfibrozil and vehicle-treated donor animals were comparable (Table 2).

**Table 1:** Biochemical parameters of gemfibrozil and vehicle-treated non-transplanted group.

Variable	nHTX	GEM-nHTX	p
Albumine (g/l)	39.1 ± 0.3	34.6 ± 0.2	<0.001
Alkaline phosphatase (U/l)	33.2 ± 2.7	41.3 ± 5.6	0.221
GPT (U/l)	35.6 ± 1.6	34.4 ± 0.6	0.482
GOT (U/l)	57.6 ± 2.1	55.8 ± 2.1	0.544
Direct bilirubine (µmol/l)	0.6 ± 0.1	0.4 ± 0.1	0.19
Total bilirubine (µmol/l)	1.3 ± 0.2	0.7 ± 0.1	0.036
Total cholesterol (mmol/l)	2.2 ± 0.1	1.7 ± 0.1	0.024
CK (U/l)	162.5 ± 8.4	163 ± 6.2	0.962
Creatinine (µmol/l)	30.1 ± 1.7	20.5 ± 1.0	<0.001
HDL cholesterol (mmol/l)	2.0 ± 0.1	1.6 ± 0.1	0.004
LDL cholesterol (mmol/l)	0.4 ± 0.1	0.3 ± 0.1	0.039
LDH (U/l)	137.2 ± 16.7	137.5 ± 16.5	0.991
Triglyceride (mmol/l)	0.5 ± 0.1	1 ± 0.1	0.014
Carbamide (mmol/l)	5.4 ± 0.3	5.7 ± 0.3	0.697

The results of biochemical parameters of gemfibrozil- and vehicle-treated non-transplanted groups are shown. The following parameters were studied: albumine, alkaline phophatase, glutamate-pyruvate transaminase (GPT), glutamate oxaloacetate transaminase (GOT), direct and total bilirubine, total cholesterol, creatine kinase (CK), creatinine, high and low-density lipoprotein-cholesterol (HDL, LDL), lactate dehydrogenase (LDH), triglyceride and carbamide. Study groups: gemfibrozil-control group (GEM-nHTX), control-group (nHTX).

**Table 2:** Hemodynamic parameters of gemfibrozil and vehicle-treated non-transplanted group.

Hemodynamic variables	GEM-nHTX	nHTX	p
Arterial systolic pressure (mmHg)	119.7 ± 3.6	115.4 ± 2.7	0.3739
Arterial diastolic pressure (mmHg)	91.3 ± 3.7	86.5 ± 3.4	0.3620
Mean Arterial Pressure (mmHg)	103.8 ± 4.2	100.5 ± 3.4	0.5488
Body weight (g)	306 ± 7.3	317.3 ± 6.6	0.2707
Heart rate (1/min)	363.3 ± 12.6	370.1 ± 8.5	0.6653
Left ventricular end-systolic pressure (mmHg)	116.9 ± 5.9	113.6 ± 4.0	0.6607
Left ventricular end-diastolic pressure (mmHg)	6.6 ± 0.5	7.8 ± 0.8	0.2446
Left ventricular dP/dt_max_ (mmHg/s)	7269.5 ± 178.8	7947.1 ± 287.1	0.0630
Left ventricular dP/dt_min_ (mmHg/s)	−9342 ± 307.7	−9052 ± 466.6	0.6237

The results of hemodynamic investigation of gemfibrozil- and vehicle-treated non-transplanted groups are shown. Study groups: gemfibrozil-control group (GEM-nHTX), control-group (nHTX).

### Hemodynamic parameters and coronary vascular function in the graft

**Figure 1:**
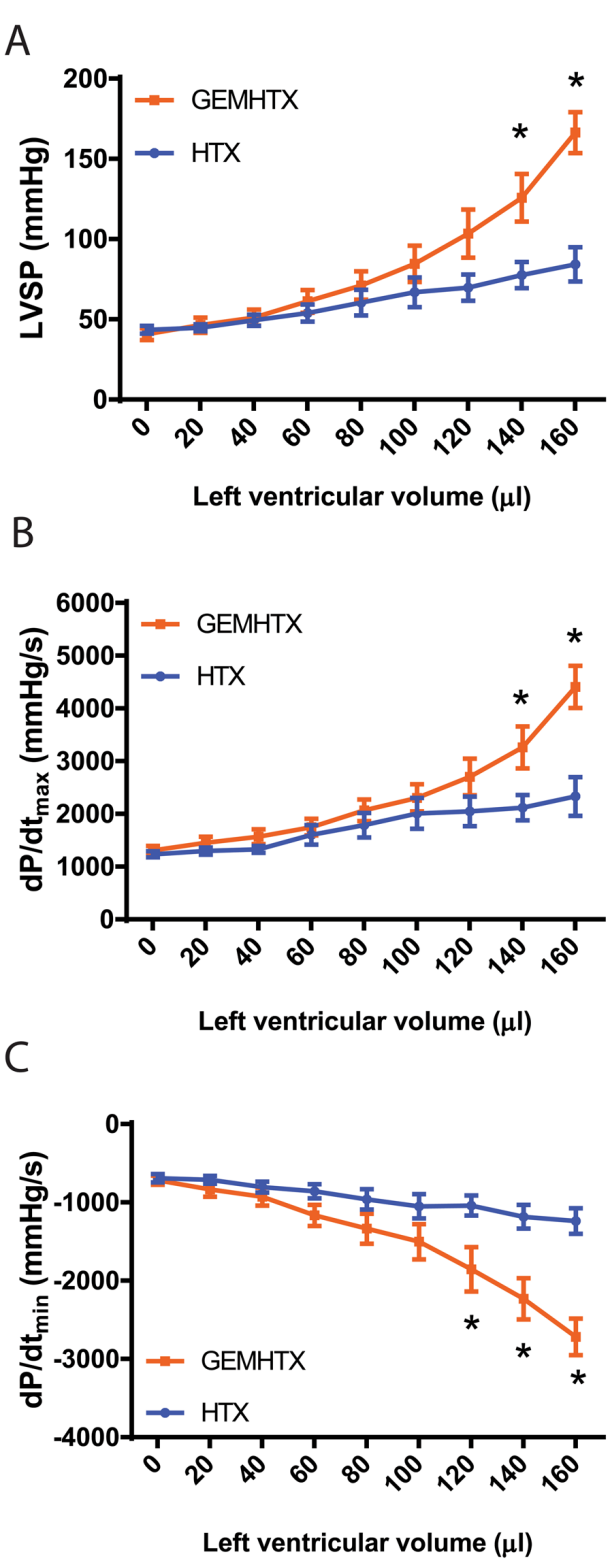
Gemfibrozil improves left ventricular systolic and diastolic function of the graft. (**A**) Maximal left ventricular systolic pressure (LVSP), (**B**) Maximal slope of systolic pressure increment (dP/dt_max_), and (**C**) diastolic pressure decrement (dP/dt_min_) are shown at different LV (balloon) volumes. Groups: transplant-control group (HTX), gemfibrozil + transplant-group (GEM-HTX). *P < 0.05 vs. HTX.

**Figure 2:**
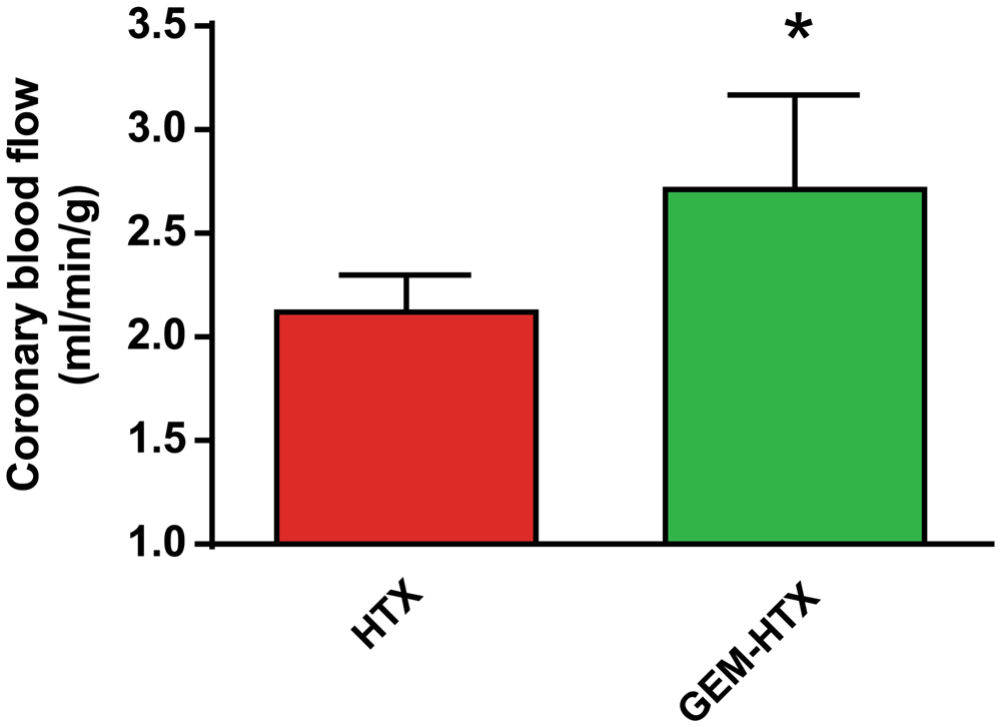
Coronary blood flow measurements. Groups: transplant-control group (HTX), gemfibrozil + transplant-group (GEM-HTX). *P < 0.05 vs. HTX.

After transplantation, increasing LV balloon volumes (‘preload’) resulted in elevated LVSP and dP/dt_max_, which were both found to be significantly increased at the largest preload volumes used in GEM-HTX compared to HTX (Fig. 1A,B). Moreover, gemfibrozil treatment led to a similar improvement in diastolic function at higher preload volumes, resulting in significantly (p < 0.05) increased dP/dt_min_ values compared to HTX, reflecting better myocardial relaxation (Fig. 1C). Coronary blood flow was also significantly (p < 0.05) increased after 1 h of reperfusion in GEM-HTX compared to the HTX group (Fig. 2), confirming significantly better coronary endothelial function in our GEM-HTX animals.

### Myocardial gene expression

**Figure 3:**
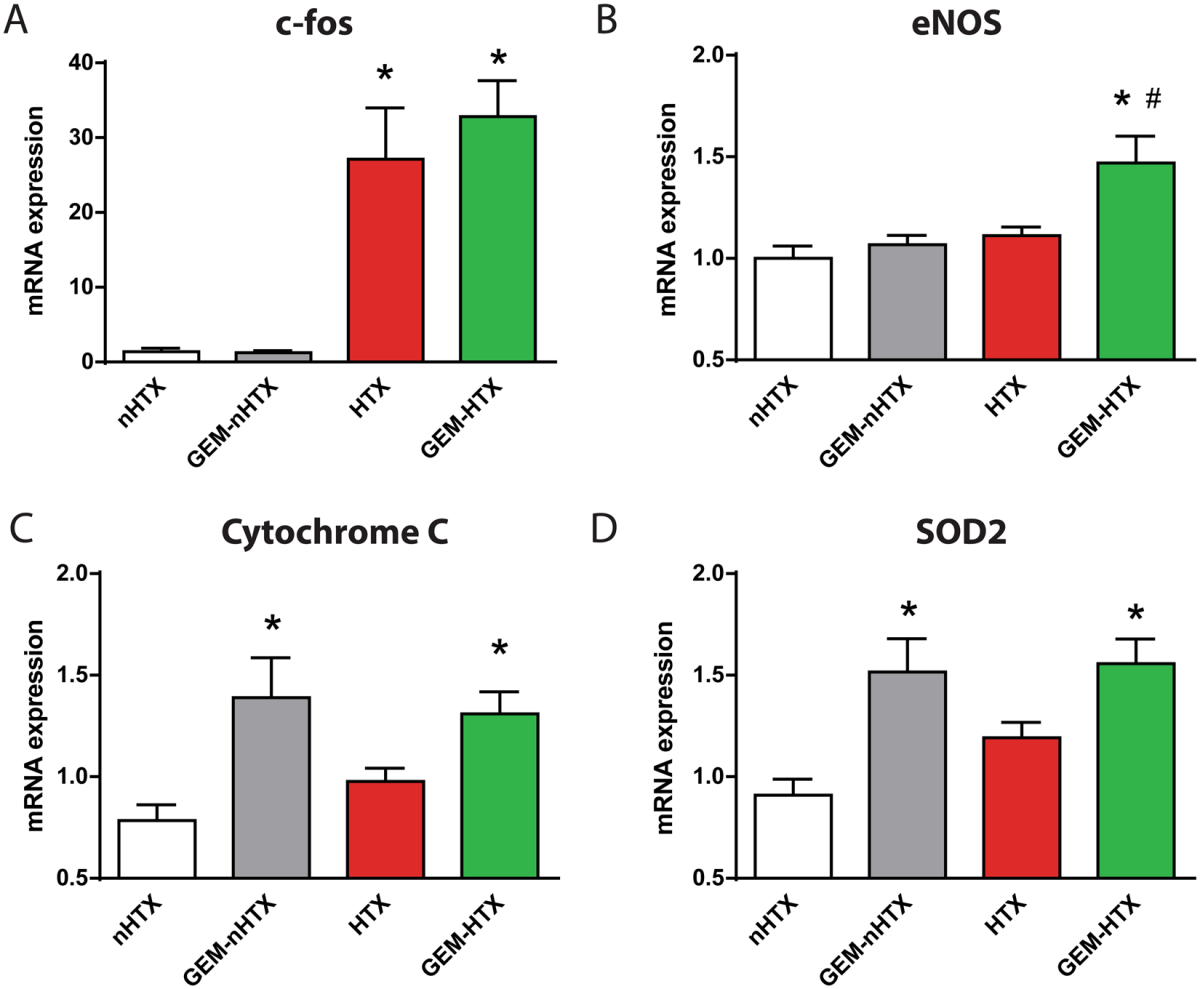
Myocardial gene expression. Myocardial mRNA expression of (**A**) c-fos, (**B**) endothelial nitrite oxide synthase (eNOS), (**C**) cytochrome C (**D**) superoxide dismutase (SOD)-2. Groups: control-group (nHTX), gemfibrozil-control group (GEM-nHTX), transplant-control group (HTX), gemfibrozil + transplant-group (GEM-HTX). *p < 0.05 vs. nHTX, ^#^p < 0.05 vs. HTX.

Quantitative RT-PCR from myocardial RNA extracts revealed that relative mRNA-expression for protooncogene c-fos was significantly upregulated in the gemfibrozil and vehicle-treated transplant group (Fig. 3A). eNOS myocardial mRNA expression was significantly elevated in the GEM-HTX group (Fig. 3B). Pharmacological preconditioning with GEM resulted in a significant increase of the mRNA expression for cytochrome C and for the antioxidant enzyme SOD2 (Fig. 3C,D).

### Myocardial protein expression

**Figure 4:**
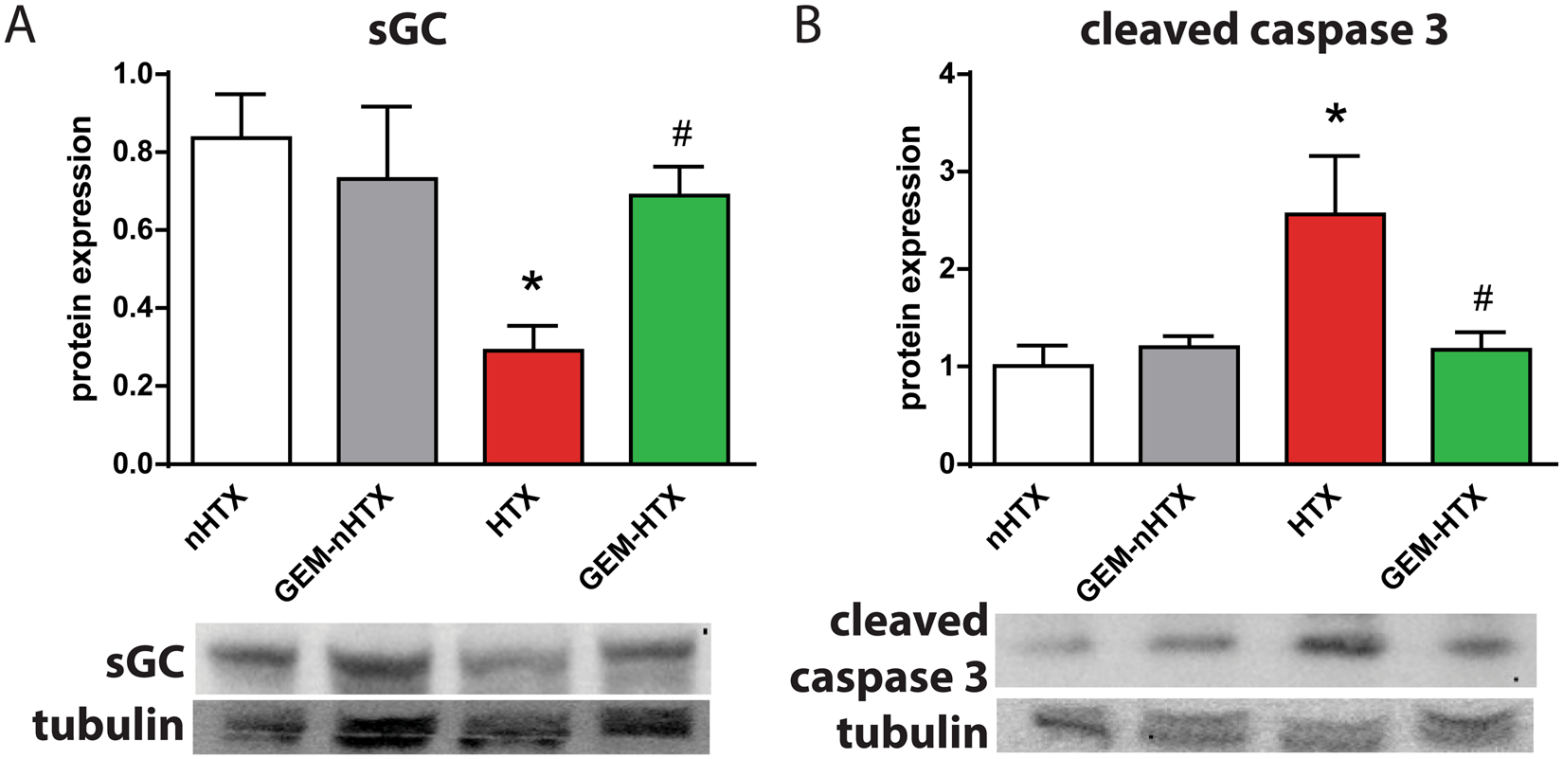
Protein expression of soluble guanylate cyclase (sGC) and cleaved caspase 3. The protein level of (**A**) sGC and (**B**) cleaved caspase 3 are shown. Groups: control-group (nHTX), gemfibrozil-control group (GEM-nHTX), transplant-control group (HTX), gemfibrozil + transplant-group (GEM-HTX). *p < 0.05 vs. nHTX, ^#^p < 0.05 vs. HTX.

Protein expression of sGC β1 was significantly reduced in the vehicle-treated transplant group whereas gemfibrozil treatment restored the enzyme’s protein level near to physiological values (Fig. 4A). We detected elevated cleaved caspase-3 protein expression in the control transplant group, whereas the application of gemfibrozil significantly reduced the expression of cleaved caspase-3 (Fig. 4B).

### Histopathology

**Figure 5:**
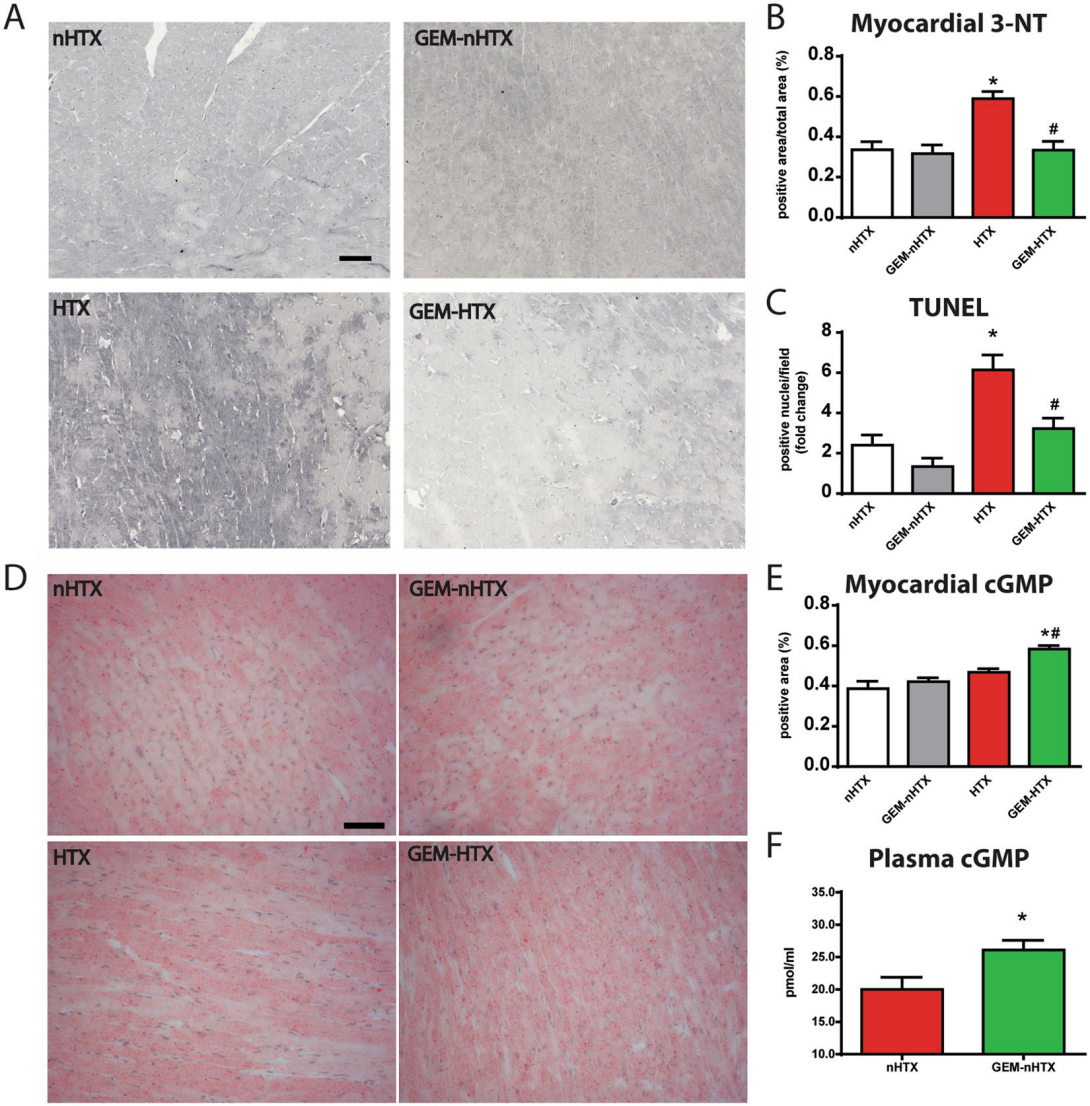
Histological analysis and plasma cGMP analysis. (**A**) Representative images of nitrotyrosine immunohistochemistry. Magnification: 200x, Marker: 50 µm (**B**) Quantification of nitrotyrosine immunohistochemistry. (**C**) Quantification of TUNEL-positive cardiomyocyte nuclei. (**D**) Representative images and quantification (**E**) of myocardial cGMP immunohistochemistry. Magnification: 200x, Marker: 50 µm (**F**) Quantification of plasma cGMP. Groups: control-group (nHTX), gemfibrozil-control group (GEM-nHTX), transplant-control group (HTX), gemfibrozil + transplant-group (GEM-HTX). *p < 0.05 vs. nHTX, ^#^p < 0.05 vs. HTX.

The HTX group was associated with increased 3-NT immunoreactivity in LV myocardium referring to pronounced nitro-oxidative stress, which was significantly (p < 0.05) prevented by gemfibrozil (Fig. 5A,B). The number of TUNEL-positive nuclei was significantly (p < 0.05) increased in the myocardium after transplantation referring to pronounced DNA fragmentation (Fig. 5C), GEM succesfully reduced the ischemia/reperfusion injury-induced DNA-strand breaks in HTX represented by markedly reduced TUNEL positivity (Fig. 5C).

### Myocardial and plasma cGMP levels in the donors

**Figure 6:**
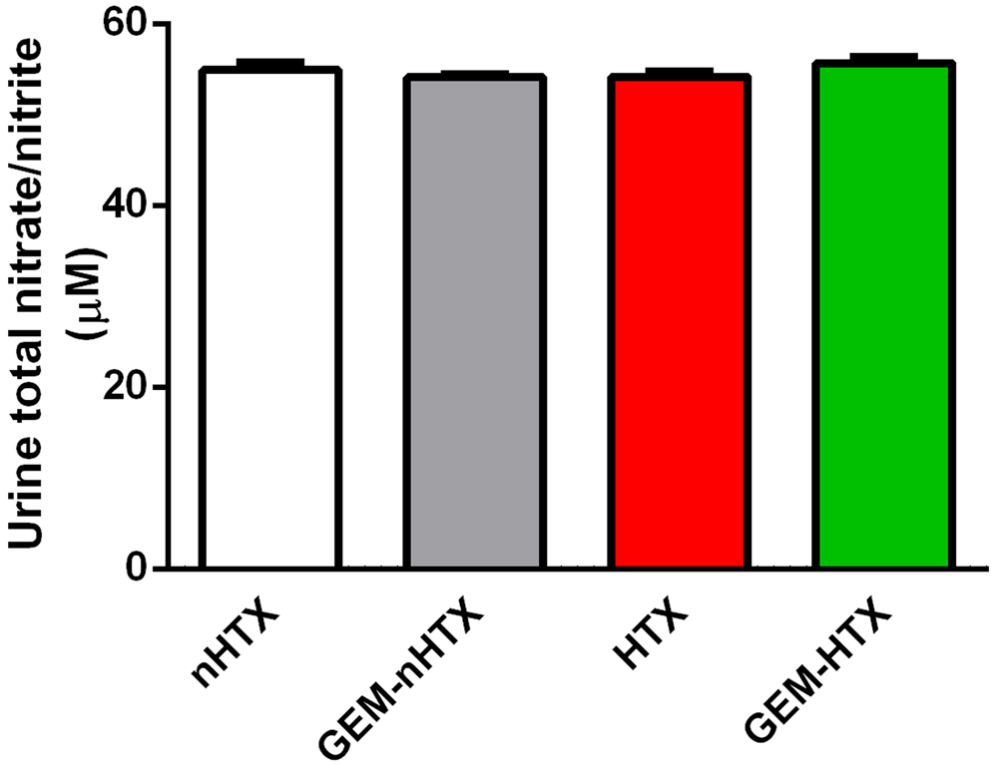
Quantification of total nitrate/nitrite concentration in urine samples. Groups: control-group (nHTX), gemfibrozil-control group (GEM-nHTX), transplant-control group (HTX), gemfibrozil + transplant-group (GEM-HTX).

Semiquantitative analysis of myocardial cGMP levels has shown that there was significantly (p < 0.05) more cGMP in the myocardium of GEM-HTX animals than in the other groups (Fig. 5D,E). Similarly, gemfibrozil preconditioning activated sGC and thus increased the plasma cGMP levels in GEM-nHTX (Fig. 5F). The increased cGMP level does not seem to be the consequence of a difference in NO production in these animals, as total nitrate/nitrite concentration of urine samples were not different (Fig. 6).

### Platelet activation and recruitment of leukocytes in the myocardium

**Figure 7:**
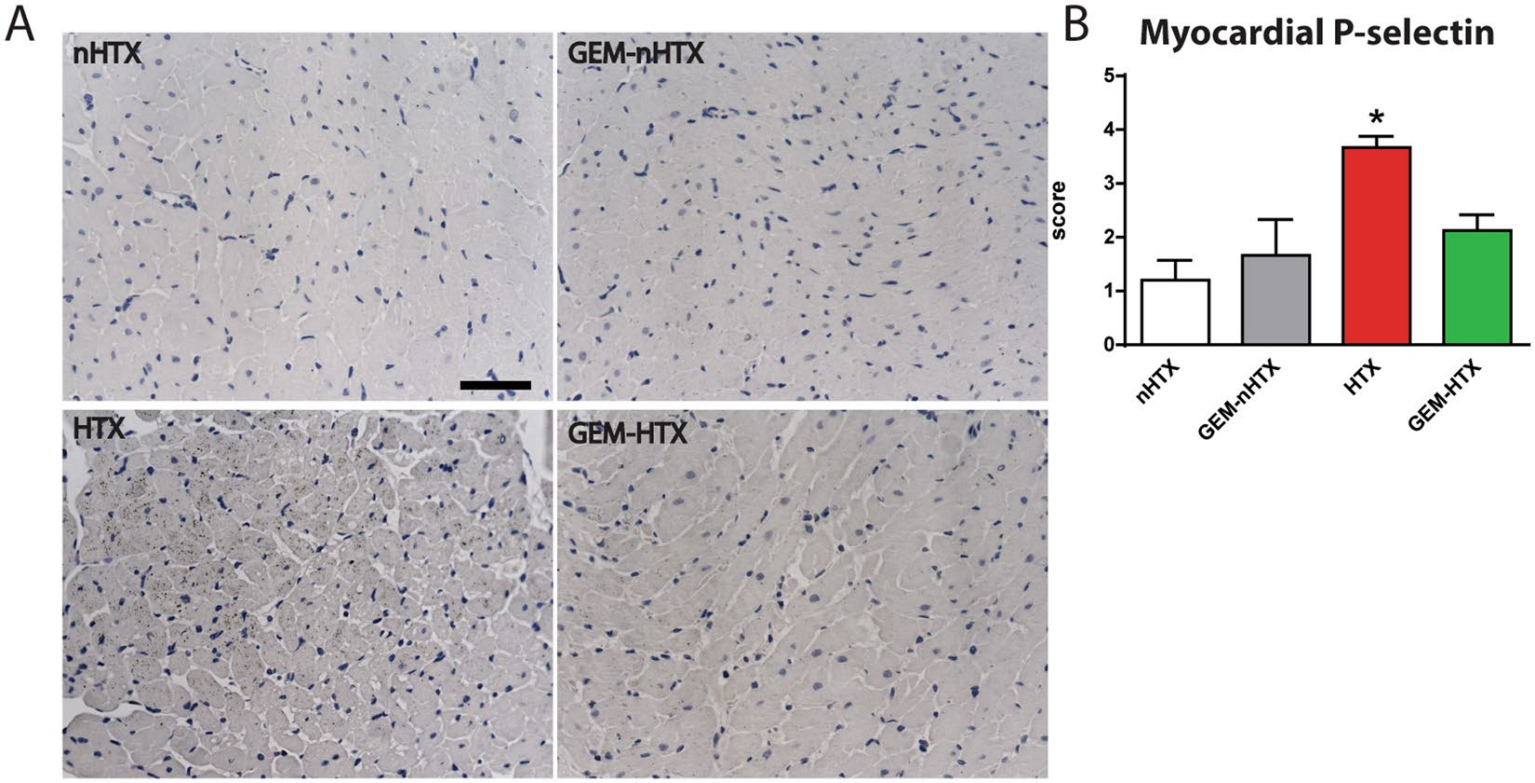
Histological analysis of P-selectin immunohistochemistry. (**A**) Representative images of myocardial P-selectin immunohistochemistry. Magnification: 200x, Marker: 50 µm (**B**) Quantification of myocardial P-selectin immunohistochemistry. Groups: control-group (nHTX), gemfibrozil-control group (GEM-nHTX), transplant-control group (HTX), gemfibrozil + transplant-group (GEM-HTX). *p < 0.05 vs. nHTX, ^#^p < 0.05 vs. HTX.

Essentially no platelets or neutrophils were detected in the myocardial tissue of the non-transplanted groups (Fig. 7A). After 1 hour of cold ischemic time and reperfusion, increased myocardial P-selectin immunoreactivity was detected reflecting platelet activation and leukocyte recruitment in the vasculature of previously ischemic myocardium (Fig. 7A,B). However, GEM treatment prevented the increase of P-selectin immunoreactivity in the myocardium after transplantation (Fig. 7B).

## Discussion

Our present study is the first to investigate the effect of gemfibrozil on global myocardial ischemia/reperfusion injury in an *in vivo* model of heterotopic rat HTX. Our results show that preconditioning of the donor heart with gemfibrozil improves post-transplant systolic and diastolic LV function and increases CBF by attenuating ischemia/reperfusion injury of the myocardium.

The gold standard therapy of terminal heart failure is HTX, however the need for donors is constantly increasing^18^. Due to the long ischemic time of the donor heart, the chance of primary graft failure is higher in the early post-operative period. As part of the ischemia/reperfusion injury-related inflammatory response, activated neutrophils release a variety of cytotoxic substances such as ROS and proteases. Moreover, they activate monocytes/macrophages that are the main source of inflammatory cytokines in the ischemic heart. Inflammatory cytokines released from activated leukocytes directly mediate vascular endothelial dysfunction with subsequent myocardial injury and the deterioration of NO-cGMP signalling. Additionally, after a long ischemic transport time, the level of ROS massively increases during reperfusion and the increased superoxide production scavenges the small amount of remaining NO contributing significantly to the decreased NO bioavailability. The NO-sGC-cGMP-PKG pathway has important physiological role in the regulation of cardiac function including coronary vasodilation, inhibition of inflammatory pathways, attenuation of oxidative stress and in the modulation of cardiac contractility^19,20^. Recent literature data suggest that restoration of cGMP-PKG signalling has potential cardioprotective effects in various cardiac diseases^14–16^ including HTX-associated ischemia/reperfusion injury^1^.

Oxidative stress triggers a decrease in sGC expression and activity^21^. Furthermore, oxidative stress is associated with increased expression and activity of PDE-5^22^ which leads to the imbalance in cGMP synthesis and degradation. This results in lower cGMP-levels and thus in impaired regulation of cGMP-PKG signalling pathway^23^. However, a novel class of drugs, the sGC activators might overcome this issue by preserving the structure, function and activity of sGC in nitro-oxidative stress^23^. Although sGC activation is a promising way to go, these compounds have not been approved for clinical use yet. The widely used fibrate gemfibrozil has been recently described as a NO- and haem-independent activator of sGC^13^. Sharina *et al*. suggested that the mechanism of action of gemfibrozil was similar to haem-mimicking sGC-activators, however it was less potent than cinaciguat or ataciguat^13^. The advantage of gemfibrozil is that it has been approved for decades and used in the pharmacological management of hyperlipidemia^13^. According to the above findings, we have observed significantly reduced protein level of sGC in the HTX group which might reflect to the increased rate of degradation of the enzyme. Gemfibrozil treatment, however, prevented the degradation of sGC that might have resulted in preserved function of sGC in our model. Furthermore, pharmacological preconditioning with gemfibrozil was associated not only with signficantly increased plasma cGMP levels in the donor but also it increased myocardial cGMP level after HTX without affecting NO production in our animals. This phenomenon might play a key role in the reduction of ischemia/reperfusion injury of the graft. Another possible explanation for the above observed effects is that the sGC-activator gemfibrozil might improve the synthesis of different antioxidant proteins via PKG-mediated processes^24^. These events could notably contribute to vaso- and cardioprotection in oxidative injury.

Oxidative and nitrosative stress lead to the formation of peroxynitrite which is a highly reactive molecule that can disrupt enzymes and other proteins^25^. Peroxynitrite can also uncouple eNOS that becomes a dysfunctional superoxide-generating enzyme contributing to enhanced oxidative stress^26^. The myocardial antioxidant enzyme SOD2 plays a major role in the elimination of ischemia/repefusion-induced elevation of ROS^27^. In accordance with this, we observed significantly elevated 3-NT staining in HTX, reflecting increased nitro-oxidative stress. However, SOD2 mRNA expression increased in the gemfibrozil treated groups, which process might have contributed to the antioxidant effects of the drug. Additionally, the sGC-derived cGMP activates PKG that in turn leads to the opening of the mitochondrial ATP-sensitive potassium channels^28^ and to increased potassium-influx into the mitochondrion. This mechanism enables protons to be pumped out for the formation of H^+^ eletrochemical gradient and thereby it increases mitochondrial ATP-synthesis^29^. The above mechanism might have played a role in the cardioprotective effects of sGC activation by gemfibrozil by the preservation of ATP-synthesis in ischemia/reperfusion injury. Moreover, myocardial cytochrome-c (terminal member of the mitochondrial electron transport chain) was upregulated in the gemfibrozil-treated transplanted group which might indicate that gemfibrozil enhances mitochondrial ATP-production.

As a result of increased nitro-oxidative stress and the disturbed myocardial energy balance (including ATP depletion) after ischemia/reperfusion, myocardial cell apoptosis and necrosis may occur^30^. In accordance with this, the modulation of the ATP degradation pathways at different levels is an important strategy to reduce tissue injury in ischemia/reperfusion^31^. Additionally, c-fos, a transcription factor of the activator protein-1 family has been linked to apoptosis^32^. Furthermore, caspase 3 has a central role in the execution phase of cell death as it is responsible for chromatin condensation and DNA fragmentation^26^. Agosto M. et colleagues described that in ST-segment elevation myocardial infarction (STEMI) the severity of the myocardial injury was correlated with the caspase 3 (p17 fragment) serum level^33^. Our findings are in concordance with the above data, since we observed increased number of TUNEL positive cardiomyocyte nuclei (marker of increased DNA fragmentation), significantly elevated level of cleaved caspase 3 (marker of caspase 3 activation) and the overexpression of c-fos transcription factor in HTX. The sGC activator gemfibrozil, however, has been shown to have anti-apoptotic properties in the donor heart by reducing TUNEL positivity and caspase 3 activation. Interestingly, gemfibrozil had no effect on c-fos mRNA level which might have been a result of the relative short reperfusion phase.

Heterotopic HTX was used in our study to simulate the clinical conditions of HTX with whole blood reperfusion. This method allows an observation time of 60 minutes. Based on literature data, we determined the reperfusion time to 1 h, where the ischemia/reperfusion injury is the most pronounced with subsequent functional deterioration of the graft^17^.

Although heterotopically transplanted hearts in our model beat in an unloaded fashion (no preload), cardiac pump function can be reliably assessed with the help of a ballon catheter inserted into the LV of the graft. Both systolic and diastolic performance of the LV can be investigated by registering LVSP, dP/dt_max_, and dP/dt_min_, respectively, at different LV (balloon) volumes. The increased slope of the relations between these parameters and LV volume demonstrate preserved systolic (contractility) and diastolic function (active relaxation) in the GEM-HTX group when compared to controls.

Moreover, GEM treatment resulted in significant improvement of CBF in the treated-HTX group which might reflect the vasoprotective properties of the drug. We hypothesize that the activation of sGC^34^ by gemfibrozil and the subsequently enhanced cGMP-PKG signalling promotes multiple phosphorylation of intracellular targets, reduce endothelial injury^35^ and can lower the cellular Ca^2+^-concentrations that might contribute to vasodilation and vasoprotection^36^. This vasodilatative effect of gemfibrozil could be responsible for the elevated CBF and might lead to a rapid recovery of the stunned myocardium during reperfusion. Additionally, it has been described that GEM succesfully reduced the incidence of cardiac allograft vasculopathy after HTX in adults by lowering endothelial damage^37^. Moreover, sGC activation and the possible eNOS reactivation in the coronaries (indicated by increased eNOS mRNA expression) and NO production might contributed synergistically to the decreased amount of nitro-oxidative stress in our model. Consequently, we observed improved systolic and diastolic function of the graft after HTX.

In accordance with previous works with other sGC-activators^1^ we found that GEM did not affect cardiac function of healthy control rats. Thus, the improved cardiac function seen in the GEM-HTX group is a specific phenomenon, reflecting a protection against the ischemia/reperfusion-associated impairment of myocardial performance, rather than the consequence of some non-specific direct cardiac effects of GEM. Although fibrates are described to have potential harmful side effects^38^, our biochemical results showed no signs of liver, kidney or muscle damage in the GEM treated healthy rats. Thus, gemfibrozil could become a safe and useful tool for the management of cardiac donors without having substantial side effects on other organs.

Microvascular obstruction is considered to be one of the major manifestations of myocardial ischemia-reperfusion injury and is associated with enhanced leukocyte invasion^39^. P-selectin, a transmembrane adhesion molecule (glycoprotein), is constitutively stored in granules of endothelial cells and platelets but becomes exposed under pathological circumstances^40^. P-selectin plays a pivotal role during ischemia-reperfusion injury by enhancing leukocyte invasion. Indeed, a previous study found that blockade of P-selectin protected against acute renal failure following ischemia-reperfusion injury^41^. In line with these findings, here we document a significantly higher myocardial P-selectin immunoreactivity in non-treated grafts. However, gemfibrozil pretreatment was associated with a P-selectin level that was comparable to that of control hearts (i.e. non-transplant groups, which did not suffer global cardiac ischemia/reperfusion injury). This may highlight the potent anti-inflammatory effect of the sGC activator gemfibrozil, which could substantially contribute to the cardioprotective effect of the drug in the setting of ischemia/reperfusion injury.

Our study have several limitations. First the donor rat hearts are implanted to recipients with ‘healthy’ circulatory system. In that case, the CBF of the graft becomes elevated thus it might partly be responsible for the improved myocardial protection after ischemia/reperfusion injury. Besides, ischemia/reperfusion injury is a reversible phenomenon in this model and it allows a fast recovery, making it difficult to find differences between experimental groups after a certain time of reperfusion. Additionally, the dose of administration of gemfibrozil might be different in a clinical setup due to the differences of pharmacokinetic and pharmacodynamic properties in rats and in humans.

To conclude, gemfibrozil treatment improves donor heart function in an experimental model of HTX. The potential sGC activator properties of gemfibrozil might be in the background of its cardioprotective effects. These findings show that pharmacological preconditioning with gemfibrozil could be a promising option to reduce ischemia/reperfusion injury and to increase the ischemic time in order to gain enough donor organs for cardiac transplantation.

## Methods

### Animals

Male Lewis rats (250–350 g; Charles River, Germany) were housed in a room at 22 ± 2 °C under 12-h light/dark cycles and were fed a standard laboratory rat diet and water *ad libitum*. The rats were acclimatized for at least 1 week before experiments. All animals received humane care in compliance with the “Principles of Laboratory Animal Care” formulated by the National Society for Medical Research and the “Guide for the Care and Use of Laboratory Animals” prepared by the Institute of Laboratory Animal Resources and used by the National Institutes of Health (Guide for the Care and Use of Laboratory Animals, published by the US National Institutes of Health [Eighth Edition] the National Academy Press. 2011 and directive 2010/63/EU of the European parliament and of the council on the protection of animals used for scientific purposes. Official Journal of the European Union. 2010). All procedures and handling of the animals during the study were reviewed and approved by the Ethical Committee of Hungary for Animal Experimentation.

### Experimental groups

Rats were randomly divided into four groups: (1) control-group (nHTX, n = 8): donor rats received methylcellulose vehicle and hearts were not transplanted (2) gemfibrozil-control group (GEM-nHTX, n = 8): donor rats received gemfibrozil and hearts were not transplanted (3) transplant-control group (HTX): donor rats (n = 8) received methylcellulose vehicle, then hearts were transplanted into the recipients (n = 8) and (4) gemfibrozil + transplant-group (GEM-HTX): donor rats (n = 8) received gemfibrozil, then hearts were transplanted into the recipients (n = 8). Donor rats were preoperatively treated orally with vehicle or gemfibrozil while recipient rats received no treatment.

### Drug application

GEM was purchased from Sigma-Aldrich (Taufkirchen, Germany), suspended in 1% methylcellulose solution vehicle and administered via oral gavage at the dose of 150 mg/kg body weight (BW). The application started two days before explantation twice a day (8:00 AM and 08:00 PM) and one dose an hour before the procedure. This dose and administration method have been determined according to the pharmacokinetic and –dynamic properties^42^ of gemfibrozil as well as to the results of previous rodent and human experiments^13,43,44^.

### Rat model of heart transplantation

Rat model of heart transplantation was performed as described previously^17,45^. Transplantations were performed in isogenic Lewis to Lewis rats in order to avoid possible organ rejection. Briefly, the donor rats were anaesthetized with isoflurane and heparinized (25000 IU iv). A bilateral thoracotomy was performed to expose the heart. After cardiac arrest the superior and inferior caval veins and the pulmonary veins were tied en masse with a suture and the heart was excised with the aortic arch for future measurement of the coronary blood flow (CBF). After excision hearts were stored in cold cardioplegic solution (Custodiol, 4 °C, Dr. Franz Köhler Chemie GmbH, Bensheim, Germany). The recipient rats were anaesthetized with isoflurane and then heparinized (400 IU/kg iv) and the body temperature was maintained at 37 °C on a heating pad. Approximately two-centimeter segments of the infrarenal aorta and the caval vein were isolated and occluded by small-vessel forceps. The aorta and the pulmonary artery of the donor heart were anastomosed end to side to the abdominal aorta and the vena cava of the recipient rat, respectively. The duration of the implantation was standardized at 1 h (ischemic period) to minimize variability between experiments. After the completion of the anastomoses, heparin was antagonized with protamin (400 IU/kg iv.) and the occlusion was released and the heart was then reperfused with blood *in situ* for 60 minutes.

### Biochemical measurements

After hemodynamic measurements of the non-transplanted groups, blood samples were collected from the inferior vena cava in tubes pre-rinsed with EDTA. Plasma albumine, alkaline phosphatase, glutamate-pyruvate transaminase (GPT), glutamate oxaloacetate transaminase (GOT), high and low-density lipoprotein-cholesterol (HDL, LDL), total cholesterol, triglyceride, direct and total bilirubine, carbamide, creatinine, creatine kinase (CK) and lactate dehydrogenase (LDH) were measured by automated clinical laboratory assays on a Cobas Integra 400 (Roche Diagnostics, Mannheim, Germany) autoanalyzer.

Plasma cGMP levels were determined from the non-transplanted groups by enzyme immunoassay (EIA) using a commercial kit (Amersham cGMP EIA Biotrak System, GE Healthcare, Buckinghamshire, UK). Total nitrate/nitrite concentration (a marker of whole body NO production) was measured from urine samples using a colorimetric kit (Cayman Chemical, Ann Arbor, MI, USA). We followed the protocols supplied by the producers.

### Functional measurements in the donor

We compared the effect of the treatment on the hemodynamic parameters of the donor heart before explantation as follows. Invasive hemodynamic measurements were carried out with a 2F microtip pressure-conductance microcatheter (SPR-838, Millar Instruments, Houston, TX, USA) with modifications as described previously^46,47^. Animals were anesthetized with isoflurane (1–2%). Rats were placed on heating pads to maintain core temperature at 37 °C. The left external jugular vein was cannulated with a polyethylene catheter for fluid administration. Firstly, mean arterial blood pressure (MAP) and heart rate (HR) were recorded. Thereafter, the catheter was advanced into the left ventricle (LV) under pressure control. Signals were recorded at a sampling rate of 1000 samples/s using a pressure-volume (P-V) conductance system (MPVS-Ultra, Millar Instruments) and the PowerLab 16/30 data acquisition system (AD Instruments, Colorado Springs, CO, USA). We used a special P-V analysis programme (PVAN, Millar Instruments) to calculate mean arterial pressure (MAP), maximal LV end-systolic pressure, LV end-diastolic pressure, maximal slope of systolic pressure increment (dP/dt_max_) and diastolic pressure decrement (dP/dt_min_).

### Functional measurements in the graft

One hour after transplantation a 3F latex balloon catheter (Edwards Lifesciences Corporation, Irvine, CA, USA) was introduced into the left ventricle via the apex to determine maximal left ventricular (LV) systolic pressure (LVSP), maximal slope of the systolic pressure increment (dP/dt_max_) and diastolic pressure decrement (dP/dt_min_) and heart rate (HR) by a Millar micromanometer (SPR-838, Millar Instruments) at different LV volumes (20–160 µl). From these data LV pressure-volume relationships were constructed. Coronary blood flow (CBF) of the graft was measured by an ultrasonic flow meter (Transonic Systems Inc., Ithaca,USA) mounted on the donor ascending aorta.

### Quantitative real-time polymerase chain-reaction (PCR)

Frozen LV samples were homogenized, total RNA was isolated as described previously^46^. Reverse transcription was performed and cDNA samples were amplified on the StepOnePlus™ Real-Time PCR System (Applied Biosystems, Foster City, CA, USA) using TaqMan® Universal PCR MasterMix and TaqMan® Gene Expression Assays (Applied Biosystems) for the following targets: c-fos (Rn02396759_m1) a transcription factor, cytochrome-c (Rn00470541_g1) a terminal member of the mitochondrial electron transport chain, members of different antioxidant systems like superoxide dismutase (SOD-2; Rn00690587_g1), and the endothelial nitrite oxide synthase (eNOS; Rn02132634_s1). Gene expression data were normalized to Ribosomal Protein L27 (RPL27; rn00821099_g1) as housekeeping gene. The mRNA expression levels were calculated using the CT comparative method (2^−ΔCT^) and adjusted to a pool of nHTX group.

### Western blot analysis

LV tissue samples were homogenized, protein concentration was determined as described previously^46^ and equal amounts of protein were separated via gel-electrophoresis. Proteins were transferred to nitrocellulose membranes. After blocking, membranes were incubated with primary antibodies against sGC (1:1000 SAB4501344, Sigma-Aldrich, Budapest, Hungary) and caspase-3 (1:2000, Cell Signaling Technology, MA, USA #9662,). After washing, membranes were incubated in horseradish peroxidase-conjugated secondary antibody. Immunoblots were developed by enhanced chemiluminescence detection. Protein band densities were quantified using GeneTools software (Syngene, Frederick, MD, USA). We have adjusted the protein band densities to α-tubulin (1:10000, sigma T5168, Sigma-Aldrich, Budapest, Hungary).

### Histology, Immunhistochemistry

Hearts were fixed in buffered paraformaldehyde solution (4%), embedded in paraffin and 5-μm thick sections were cut. Terminal deoxynucleotidyl transferase-mediated dUTP nick end-labeling (TUNEL) reaction was performed (DeadEnd™ Colorimetric TUNEL System, Promega, Mannheim, Germany) to detect DNA strand breaks. The nitro-oxidative stress marker 3-nitrotyrosine (3-NT) (#10189540, Cayman Chemical, Ann Arbor, MI, USA) was detected by immunohistochemical staining as previously described^46^ and 3-NT positive area was analyzed with Image J (NIH, Bethesda, MD, USA) software. For identification of intracellular cGMP (#ab12416, Abcam, Cambridge, UK), immunohistochemistry was performed according to a previously described method^46^.

In case of P-selectin staining, paraffin-embedded sections (5 μm) were rehydrated and incubated with 1% hydrogen peroxide. After being rinsed in PBS, the sections were incubated with blocking serum. Immunostaining was performed with the use of a mouse polyclonal antibody (Impress Reagent, Vector Laboratories, Peterborough, UK)^48^.

### Statistical analysis

Data are expressed as mean ± SEM. Normal distribution was tested by Kolmogorov-Smirnov test. In case of normal distribution, one-way ANOVA and Bonferroni post hoc test were performed. Where data showed not normal distribution Kruskal-Wallis ANOVA and Dunn’s post hoc test were done. In case of hemodynamic data in HTX two-way ANOVA with Bonferroni post hoc test was used. P < 0.05 was considered statistically significant.
